# FLT PET/CT imaging of metastatic prostate cancer patients treated with pTVG-HP DNA vaccine and pembrolizumab

**DOI:** 10.1186/s40425-019-0516-1

**Published:** 2019-01-30

**Authors:** Matthew Scarpelli, Christopher Zahm, Scott Perlman, Douglas G. McNeel, Robert Jeraj, Glenn Liu

**Affiliations:** 1 Department of Medical Physics, University of Wisconsin, 1111 Highland Ave, Madison, WI 53792 USA; 2 University of Wisconsin Carbone Cancer Center, 1111 Highland Ave, Madison, WI 53792 USA; 3 Department of Radiology, Section of Nuclear Medicine, University of Wisconsin, 600 Highland Avenue, Madison, WI 53792 USA; 4 Department of Medicine, Division of Hematology/Oncology, University of Wisconsin, 600 Highland Avenue, Madison, WI 53792 USA

**Keywords:** FLT PET, Imaging, Cell proliferation, DNA vaccine, Pembrolizumab, Prostate cancer, Clinical trial, Adverse events, Response assessment

## Abstract

**Background:**

Immunotherapy has demonstrated remarkable success in treating different cancers. Nonetheless, a large number of patients do not respond, many respond without immediate changes detectable with conventional imaging, and many have unusual immune-related adverse events that cannot be predicted in advance. In this exploratory study, we investigate how 3′-Deoxy-3’-^18^F-fluorothymidine (FLT) positron emission tomography (PET) measurements of tumor and immune cell proliferation might be utilized as biomarkers in immunotherapy.

**Methods:**

Seventeen patients with metastatic castrate resistant prostate cancer were treated with combination pTVG-HP DNA vaccine and pembrolizumab. Patients underwent baseline and 12-week FLT PET/CT scans. FLT PET standardized uptake values (SUVs) were extracted from tumors, non-metastatic lymph nodes, spleen, bone marrow, pancreas, and thyroid to quantify cell proliferation in these tissues. Regional immune cell responses to pTVG-HP DNA vaccine were assessed by comparing FLT uptake changes in vaccine draining and non-draining lymph nodes. Cox proportional hazards regression was utilized to relate FLT uptake and other clinical markers (PSA and tumor size) to progression-free survival. Area under receiver operating characteristic (AUC) curves and concordance indices were used to assess the predictive capabilities of FLT uptake.

**Results:**

Changes in FLT uptake in vaccine draining lymph nodes were significantly greater than changes in non-draining lymph nodes (*P* = 0.02), suggesting a regional immune response to vaccination. However, the changes in FLT uptake in lymph nodes were not significantly predictive of progression-free survival. Increases in tumor FLT uptake were significantly predictive of shorter progression-free survival (concordance index = 0.83, *P* < 0.01). Baseline FLT uptake in the thyroid was significantly predictive of whether or not a patient would subsequently experience a thyroid-related adverse event (AUC = 0.97, *P* < 0.01).

**Conclusions:**

FLT PET uptake was significantly predictive of progression-free survival and the occurrence of adverse events relating to thyroid function. The results suggest FLT PET imaging has potential as a biomarker in immunotherapy, providing a marker of tumor and immune responses, and as a possible means of anticipating specific immune-related adverse events.

**Trial registration:**

NCT02499835.

**Electronic supplementary material:**

The online version of this article (10.1186/s40425-019-0516-1) contains supplementary material, which is available to authorized users.

## Background

Despite the promise of immunotherapy for treating advanced cancers, a number of challenges remain. Typically, only a small fraction of patients achieve durable long lasting responses to therapy. Further, measuring tumor responses is complicated by the fact that responding patients may initially experience an increase in tumor size or seemingly develop new lesions on radiographic images [1]. These challenges generate a need for predictive and pharmacodynamic biomarkers [2, 3].

Numerous studies have explored potential biomarkers in immunotherapy. Some of the most successful biomarkers to date are those associated with response to T-cell checkpoint blockade therapies derived from tumor biopsies, such as tumor cell expression of programmed death-ligand 1 (PD-L1) or measurement of tumor mutational burden [4–6]. Biomarkers derived from tissue biopsies can be useful for aiding selection of therapy. However, due to the invasiveness of tissue biopsies, it is not feasible for all tumor sites/sizes and repeat measurements are difficult to procure [7]. Biomarkers derived from peripheral blood, such as presence of antigen-specific circulating T-cells, offer a less invasive supplement to biomarkers derived from tissue biopsies [8, 9]. While peripheral blood markers can be measured longitudinally with relative ease, they do not offer a direct assessment of tumor sites and often cannot provide organ specific information regarding immune responses.

A third class of biomarkers, those derived from medical imaging, offer a useful complement to tissue biopsies and peripheral blood sampling. Imaging biomarkers provide assessment of tumor sites and immune organs as well as enable repeat measurements to assess changes during treatment. Positron emission tomography (PET) is a medical imaging modality that enables non-invasive quantification of molecular changes occurring in vivo. A number of PET tracers are under development to image specific immune pathways and provide insight into the effects of immunotherapy [10–14]. However, a promising PET tracer for assessing response to immunotherapy is 3′-Deoxy-3’-^18^F-fluorothymidine (FLT). FLT is a radiolabeled molecular analogue of the DNA nucleoside thymidine and is preferentially taken up in proliferating cells after injection [15–20]. Imaging with FLT PET has been extensively utilized to assess changes in tumor cell proliferation during chemotherapy, radiotherapy, or tyrosine kinase inhibition in clinical studies [21–35]. In the context of immunotherapy, two prior clinical studies have demonstrated increased FLT uptake in lymphoid organs following immunotherapy that was indicative of increased immune cell proliferation [36, 37]. These prior results suggest FLT PET may be a valuable response biomarker in immunotherapy, providing assessment of tumor cell proliferation in tumors and immune cell proliferation in lymphoid organs.

This study reports on the exploratory aim of a clinical trial whose primary endpoints were to assess the safety and clinical effects of pTVG-HP DNA vaccine encoding prostatic acid phosphatase (PAP) given concurrently or sequentially with pembrolizumab in patients with metastatic, castration-resistant prostate cancer [38]. Here we describe changes in quantitative FLT PET/CT imaging that occurred during this combination therapy. The therapeutic rationale is that the pTVG-HP DNA vaccine will induce or augment therapeutic T-cells specific for the prostate tumor antigen PAP and combination treatment with a T cell checkpoint blockade (pembrolizumab) will preserve the effector function PAP-specific CD8+ T cells within the tumor microenvironment [39, 40]. We hypothesized that changes in cell proliferation, as measured by FLT PET, in lymphoid organs would be associated with pharmacodynamic effects of treatment and changes in cell proliferation in tumor sites would be associated with subsequent evidence of anti-tumor response. The results of this work demonstrate the utility of FLT PET for predicting tumor responses and immune-related adverse events during immunotherapy. The ultimate goal is to investigate the potential value of FLT PET/CT as an imaging biomarker in immunotherapy.

## Materials and methods

### Study design

This work was an exploratory endpoint of a clinical trial whose primary methodology and results are reported elsewhere [38]. Patients with metastatic castrate resistant prostate cancer were included in this study. Patients underwent treatment in one of three study arms (Additional file 1 Figure S1). In study Arm 1 pTVG-HP vaccine was given every 2 weeks from week 0 to week 10 and pembrolizumab was given every 3 weeks from week 0 to week 9. In study Arm 2 pTVG-HP vaccine was given every 2 weeks from week 0 to week 10 and pembrolizumab was given every 3 weeks from week 12 to week 21. In study Arm 3 both the pembrolizumab and pTVG-HP vaccine were given every 3 weeks from week 0 to week 21. The vaccine was administered intradermally (100 μg) in the left deltoid region and pembrolizumab was administered intravenously (2 mg/kg). All patients also received recombinant human granulocyte-macrophage colony-stimulating factor as a vaccine adjuvant at time of vaccine administration (208 μg). Both a baseline and follow-up FLT PET/CT scan was acquired in all treatment arms. The baseline FLT PET/CT scan was performed prior to starting treatment (within 4 weeks) and the follow-up FLT PET/CT scan was performed at the start of week 12 (± 3 days).

Patients’ serum PSA was measured at a minimum of every 6 weeks. All subjects were followed for at least 1 year, with staging CT scans of the abdomen and pelvis, and bone scintigraphy, performed every 12 weeks or as clinically indicated using Prostate Cancer Clinical Trials Working Group recommendations [41]. Changes in size of soft-tissue tumors were evaluated following Response Evaluation Criteria for Solid Tumors (RECIST) [41]. Patients came off study at the time of radiographic progression, undue toxicity, or at the discretion of the treating physician for clinical deterioration. The study protocol was reviewed and approved by all local (University of Wisconsin Human Subjects’ Review Board), and federal (FDA, NIH Recombinant DNA Advisory Committee) entities. All patients gave written informed consent for participation.

### PET/CT image acquisition and segmentation

All patients were scanned on a Discovery 710 PET/CT scanner (GE, Waukesha WI). The CT scan was used for PET attenuation correction and anatomic localization of regions of interest. A median of 345 MBq of FLT was injected (range = 210 to 363) and PET scans were started median 59.8 min post-injection (range = 59.2 to 60.7). Each PET scan was a whole body scan with 5 min per bed position (patients were scanned from thighs to mid-skull) and 700 mm axial field of view. The PET reconstruction was a 3D ordered subsets expectation maximization algorithm with an axial grid size of 192 × 192 voxels (3.64 × 3.64 mm axial voxel size), 3.27 mm slice thickness, 3 iterations, 24 subsets, and 5 mm Gaussian post filter.

To evaluate changes in immune cell proliferation, the vaccine-draining axillary lymph nodes and non-draining axillary lymph nodes were identified by a nuclear medicine physician and manually segmented using the PET and CT images. Vaccine-draining nodes were left axillary lymph nodes and non-draining nodes were right axillary lymph nodes (the vaccine was injected in the left deltoid region). The femoral bone marrow and spleen of each patient were also segmented using semi-automatic methods that utilized both the CT and PET images. Bone marrow and spleen segmentations were visually checked to ensure no metastatic disease was present within the segmentations. Tissues that were related to immune-related adverse events (pancreas and thyroid) were also manually segmented to quantify FLT uptake in these tissues. To evaluate changes in tumor cell proliferation, soft tissue metastases were identified by a nuclear medicine physician and manually segmented. Bone metastases were not analyzed due to high background FLT uptake in the proliferating bone marrow.

Standardized uptake values (SUVs) were calculated by normalizing the activity concentration in a given voxel by the ratio of injected dose divided by patient weight [42]. The average and maximum PET SUV was extracted from all segmentations (SUV_mean_ and SUV_max_, respectively). To assess tumor burden, the total tumor uptake (SUV_total_) was also extracted from tumor segmentations. In patients with multiple tumors, patient-summarized SUV metrics were derived from the individual tumor SUV metrics as follows: patient SUV_mean_ was the mean of tumor SUV_means_, patient SUV_max_ was the max of tumor SUV_maxs_, and patient SUV_total_ was the sum of tumor SUV_totals_.

### Tissue biopsy evaluation

For comparison with tumor FLT uptake, soft-tissue tumor biopsies were evaluated for changes in cell proliferation. Three patients had soft-tissue tumor biopsies (the remaining patients had bone metastases biopsies [38]). Of these 3 patients, only 1 had a successful baseline and 12-week biopsy (one patient had no tumor cells present in the follow-up biopsy, excluding it from the analysis; the other patient had a marked reduction in tumor size during treatment, making the soft-tissue tumor infeasible for biopsy upon follow-up). The biopsies of the patient who had successful baseline and 12-week biopsies, were formalin fixed paraffin embedded (FFPE) and co-stained for CD8 and Ki67 or prostate-specific membrane antigen (PSMA) and Ki67 expression using standard immunohistochemistry techniques. Slides were heated at 80°C for 30 min, deparaffinizied, and antigens retrieved using DIVA Decloaker (Biocare Medical, DV2004, Pacheco, CA) at 99 °C for 30 min. CD8 was detected with rabbit anti-human CD8 primary antibody clone SP16 (Biocare Medical, CRM 311 A), PSMA was detected with rabbit anti-human PSMA primary antibody clone D7I8E (Cell Signaling, 12815S). Both were diluted 1:100 in Van Gogh Diluent (Biocare Medical, PD902 L) and followed by anti-Rabbit-alexafluor-555 secondary antibody diluted 1:500 (Cell Signaling, 4413S). Ki67 was detected with a mouse anti-human Ki67 primary antibody clone MIB-1 (Cell Signaling, 9449S) followed by anti-mouse-alexafluor-488 (Cell Signaling, 4408S).

Immunofluorescence imaging was conducted on a Leica DMi8 and images were processed in the Fiji package of ImageJ. Images were split into RGB colors and the contrast, brightness and color balance were optimized per channel, but evenly across all areas of each image and all images of the same fluorophore. The images were then combined into a single RGB image to determine co-localization. Ten images for each CD8/Ki67 and PSMA/Ki67 were captured from randomized areas of the FFPE section. Total CD8 or PSMA positive cells were counted by hand using the cell counter function of Image J, double positive cells were similarly counted. The percentage of Ki67^+^ cells were summarized: Ki67^+^/total counted, Ki67^+^PSMA^+^/total PSMA^+^, or Ki67^+^CD8^+^/total CD8^+^ to determine the percentage of proliferating cells, percentage of proliferating tumor cells, and percentage of proliferating immune cells, respectively.

### Statistical analysis

Due to the skewed nature of PET SUV distributions [43], non-parametric statistics were used to analyze the data. Wilcoxon signed-rank tests were used to assess whether there were significant changes in PET uptake from baseline to 12 weeks. Correlations were assessed using Spearman correlations. Wilcoxon rank-sum tests were utilized to assess differences is SUV distributions between independent groups. Univariate Cox-proportional hazards regression models were used to investigate the relationship between PET uptake and radiographic progression-free survival. Patients who came off study for any reason other than radiographic progression were censored. The concordance index was used to assess the ability of PET metrics to predict progression-free survival and the hazard ratio was used to assess correlations between PET metrics and progression-free survival [44, 45]. Area under the receiver operating characteristic curves (AUCs) were used to assess the ability of FLT PET to predict adverse events [46]. Comparisons between baseline and follow-up cell counts on immunofluorescence images were made with a one-sided ANOVA followed by Bonferroni’s post-test. *P*-values less than *P* = 0.05 were considered statistically significant.

## Results

### FLT PET changes in immune organs and soft-tissue metastases

Seventeen patients completed both the baseline and 12-week FLT PET/CT scans (Table 1). The number of patients in study arms 1, 2, and 3 were 6/17 (35%), 6/17 (35%), and 5/17 (30%), respectively. The median progression-free survival time was 24 weeks (range 12 to greater than 72 weeks). No significant differences in progression-free survival time were found across the three study arms. For all segmented regions, the changes in SUV_mean_ and SUV_max_ were strongly correlated (ρ > 0.70, *P* < 0.05). Thus, the following results focus on describing changes in SUV_mean_ (the results for SUV_max_ were similar).

**Table 1:** Patient information

Patient #	Study arm	Soft tissue mets. at baseline	Bone mets. at baseline	IRAE greater than Gr 1^a^ (week of occurrence)	Progression-free survival (weeks)
1	1	Yes	Yes	None	24
2	1	Yes	Yes	None	48
3	1	No	Yes	None	24
4	1	Yes	No	None	48+
5	1	Yes	Yes	None	16
6	1	No	Yes	Hyperthyroidism (6); Hypothyroidism (12)	24
7	2	No	Yes	Hypothyroidism (36)	36+
8	2	Yes	Yes	Pancreatitis (30)	48
9	2	Yes	Yes	None	24
10	2	Yes	Yes	None	24+
11	2	No	Yes	Hyperthyroidism (18)	48
12	2	No	Yes	Adrenal Insufficiency (38)	72+
13	3	Yes	Yes	Elevated TSH (9)	36+
14	3	Yes	No	None	24
15	3	Yes	Yes	None	24
16	3	Yes	No	Hyperthyroidism (3)	12
17	3	No	Yes	None	12

^a^Immune-related adverse events that were at least possibly related to pembrolizumab and /or pTVG vaccine are listed

**Fig. 1:**
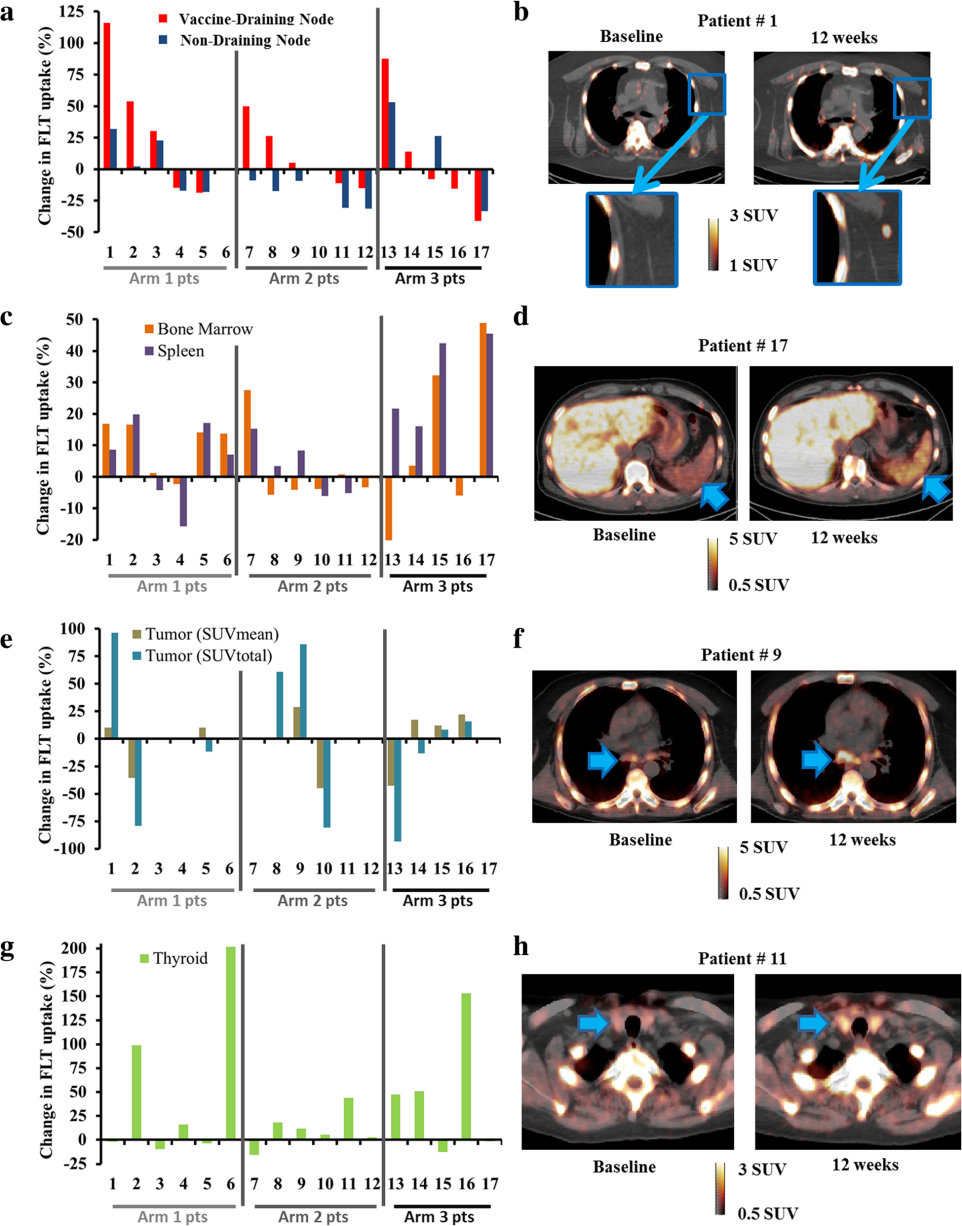
Patients are numbered the same in all inserts (also the same as in Table 1) and are ordered by study arm. **a** Changes in FLT SUV_mean_ in vaccine draining left axillary lymph nodes are shown for each patient along with changes in non-draining right axillary lymph nodes. A number of outliers with changes in left axillary lymph node SUV_mean_ greater than 50% are evident. **b** Representative FLT PET/CT slice is showing a vaccine draining left axillary lymph with elevated uptake after 12 weeks of therapy (patient #1) **c** Changes in SUV_mean_ in bone marrow and spleen. **d** Representative FLT PET/CT slice is showing increased splenic FLT uptake after 12 weeks (patient #17). **e** Changes in FLT uptake in patients with soft tissue metastases are shown for SUV_mean_ and SUV_total_. Changes in tumor SUV_mean_ and tumor SUV_total_ were significantly correlated (ρ = 0.66, *P* = 0.04). **f** Representative FLT PET/CT slice is showing metastatic mediastinal lymph nodes with visually increased FLT uptake after 12 weeks of therapy (patient #9). Following RECIST guidelines this patient had radiographically stable disease at week 12 but had subsequent disease progression upon the next radiographic follow-up at 24 weeks. **g** Changes in thyroid FLT uptake **h** Representative PET/CT slice is shown for a patient that experienced grade 2 hyperthyroidism (patient #11). The arrow indicates the position of the right thyroid lobe where visually increased FLT uptake is evident at 12 weeks. Notably, this patient had their first pembrolizumab injection 1 day prior to their 12-week PET scan

From baseline to 12 weeks, the change in SUV_mean_ of vaccine draining left axillary (sentinel) lymph nodes was significantly greater than the change in contralateral right axillary lymph nodes (median + 16%, *P* = 0.02) (Fig. 1a and b). Changes in SUV_mean_ of left axillary lymph nodes were strongly correlated with changes in right axillary lymph nodes (ρ = 0.84, *P* < 0.01). No significant differences in lymph node SUVs were evident across the study arms.

Changes in spleen and bone marrow SUV_mean_ were moderately correlated (ρ = 0.53, *P* = 0.04). The bone marrow had a median increase of 1% in SUV_mean_ (range − 20 to + 49%, *P* = 0.23). The spleen demonstrated a significant median increase of 8% in SUV_mean_ (range − 16 to + 46%, P = 0.02). Furthermore, patients in arm 3 had significantly greater increases in spleen SUV_mean_ relative to arm 1 (P = 0.04) and arm 2 (P < 0.01) (Fig. 1c and d). Neither changes in bone marrow or spleen SUV were significantly correlated with changes in lymph node SUV.

Ten patients had soft-tissue metastases that could be evaluated with FLT PET. From baseline to 12 weeks, the median tumor SUV_mean_ increased 10% (range − 45 to + 29%, *P* = 1.0) (Fig. 1e and f). Changes in tumor SUV_mean_ were not significantly correlated with changes in lymph node, bone marrow, or spleen SUVs.

From baseline to 12 weeks, the median thyroid SUV_mean_ significantly increased 12% (range − 16 to + 202%, *P* = 0.03) (Fig. 1g and h). No significant differences in the changes in thyroid SUV_mean_ were evident across the study arms. Changes in thyroid SUVs were not significantly correlated with changes in lymph node, bone marrow, spleen, or tumor SUVs.

### FLT PET and tumor responses to immunotherapy

**Fig. 2:**
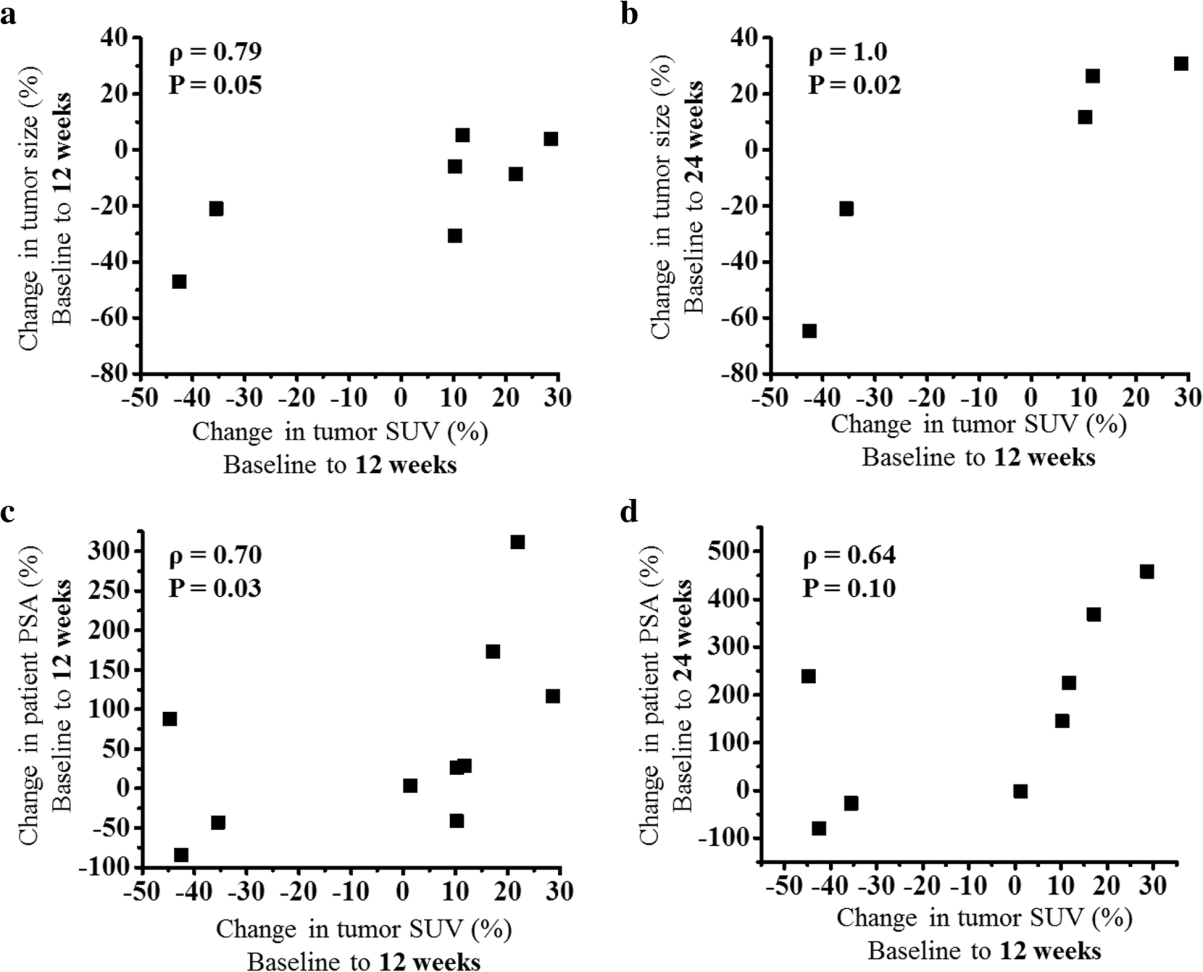
**a** Changes in tumor FLT SUV_mean_ after 12 weeks are plotted against changes in tumor size after *12 weeks*. Tumor size was measured following RECIST guidelines using a diagnostic CT scan. **b** Changes in tumor FLT SUV_mean_ after 12 weeks are plotted against changes in tumor size after *24 weeks*. **c** Changes in tumor FLT SUV_mean_ after 12 weeks are plotted against changes in PSA after *12 weeks*. **d** Changes in tumor FLT SUV_mean_ after *12 weeks* are plotted against changes in PSA after *24 weeks*. Note some patients are not included in these figures because they did not have RECIST measurable soft tissue tumors or were on study for less than 24 weeks

**Fig. 3:**
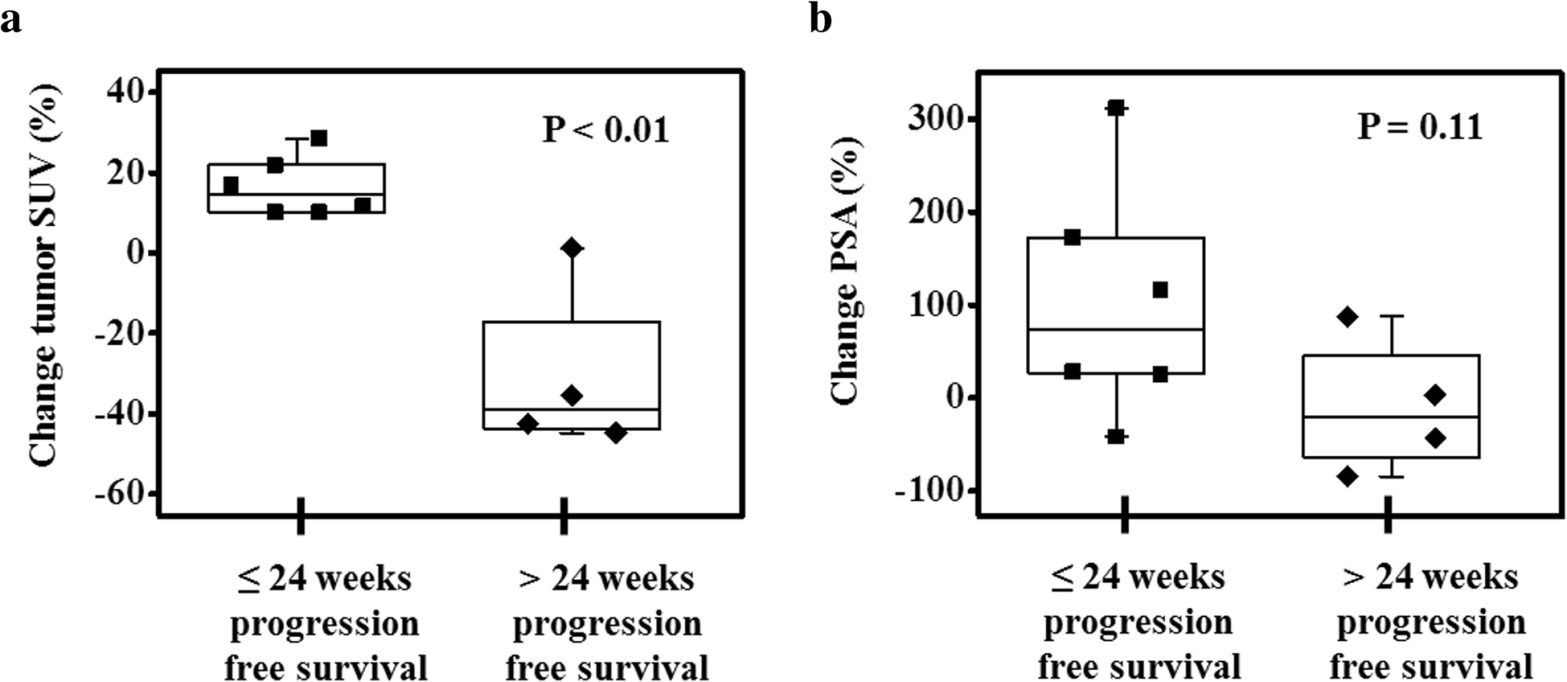
**a** Change in tumor SUV_mean_ at 12 weeks differentiated patients who had progression-free survival less than or equal to the median progression-free survival time (24 weeks) from patients who had progression-free survival greater than the median. **b** Changes in PSA levels after 12 weeks for the same set of patients as shown in insert (**a**)

Changes in tumor FLT uptake were positively correlated with changes in RECIST measurements (Fig. 2a-b) and PSA measurements (Fig. 2c-d). Changes from baseline to 12 weeks in patient PSA, RECIST measurements, and PET SUVs were included in univariate Cox proportional hazards regression models to assess their association with progression-free survival (Table 2). The strongest association was found for changes in tumor SUV_mean_ (Concordance index = 0.83, *P* < 0.01; Hazard Ratio = 3.38, *P* = 0.05). A greater increase in tumor SUV_mean_ during therapy was predictive of shorter progression-free survival. Furthermore, the change in SUV_mean_ from baseline to 12 weeks differentiated patients with progression-free survival less than or equal to the median time from those patients with progression-free survival greater than the median time (Fig. 3a). For comparison, Fig. 3b shows that greater increases in PSA were also associated with shorter progression-free survival, albeit the association was not as strong as it was for changes in tumor FLT uptake. Notably, greater increases in spleen SUV_mean_ were significantly predictive of shorter progression-free survival (Concordance index = 0.73, *P* = 0.01; Hazard Ratio = 2.14, *P* = 0.02). At baseline, only tumor SUV_mean_ and tumor SUV_total_ were significantly predictive of progression-free survival (Additional file 2 Table S1).

**Table 2:** Changes in FLT SUVs, RECIST size measurements, and PSA levels from baseline to 12 weeks were included in Cox proportional hazards regression models to assess association with progression-free survival time

	Predictor	C-index^a^	C-index *P* value	Hazard Ratio	HR P value	N^b^
Traditional markers of response	Change PSA	0.72 (0.50 to 0.94)	0.05	2.34 (1.18 to 4.62)	0.01	17 (12)
	Change soft tissue tumor size (RECIST)	0.59 (0.54 to 0.63)	< 0.01	1.78 (0.60 to 5.29)	0.30	7 (6)
FLT PET changes in lymphoid organs	Change left axillary lymph node SUV_mean_	0.70 (0.48 to 0.91)	0.07	0.89 (0.43 to 1.84)	0.75	16 (11)
	Change spleen SUV_mean_	0.73 (0.56 to 0.90)	0.01	2.14 (1.11 to 4.12)	0.02	16 (11)
	Change bone marrow SUV_mean_	0.65 (0.41 to 0.89)	0.22	1.94 (0.98 to 3.86)	0.06	17 (12)
FLT PET changes in tumors	Change tumor SUV_mean_	0.83 (0.71 to 0.95)	< 0.01	3.38 (1.01 to 11.28)	0.05	10 (8)
	Change tumor SUV_total_	0.69 (0.59 to 0.79)	< 0.01	1.53 (0.76 to 3.10)	0.24	10 (8)

^a^Concordance index (95% confidence interval shown in parenthesis)

^b^*N* = number of patients included in calculation (value in parenthesis is number of patients that were not censored)

**Fig. 4:**
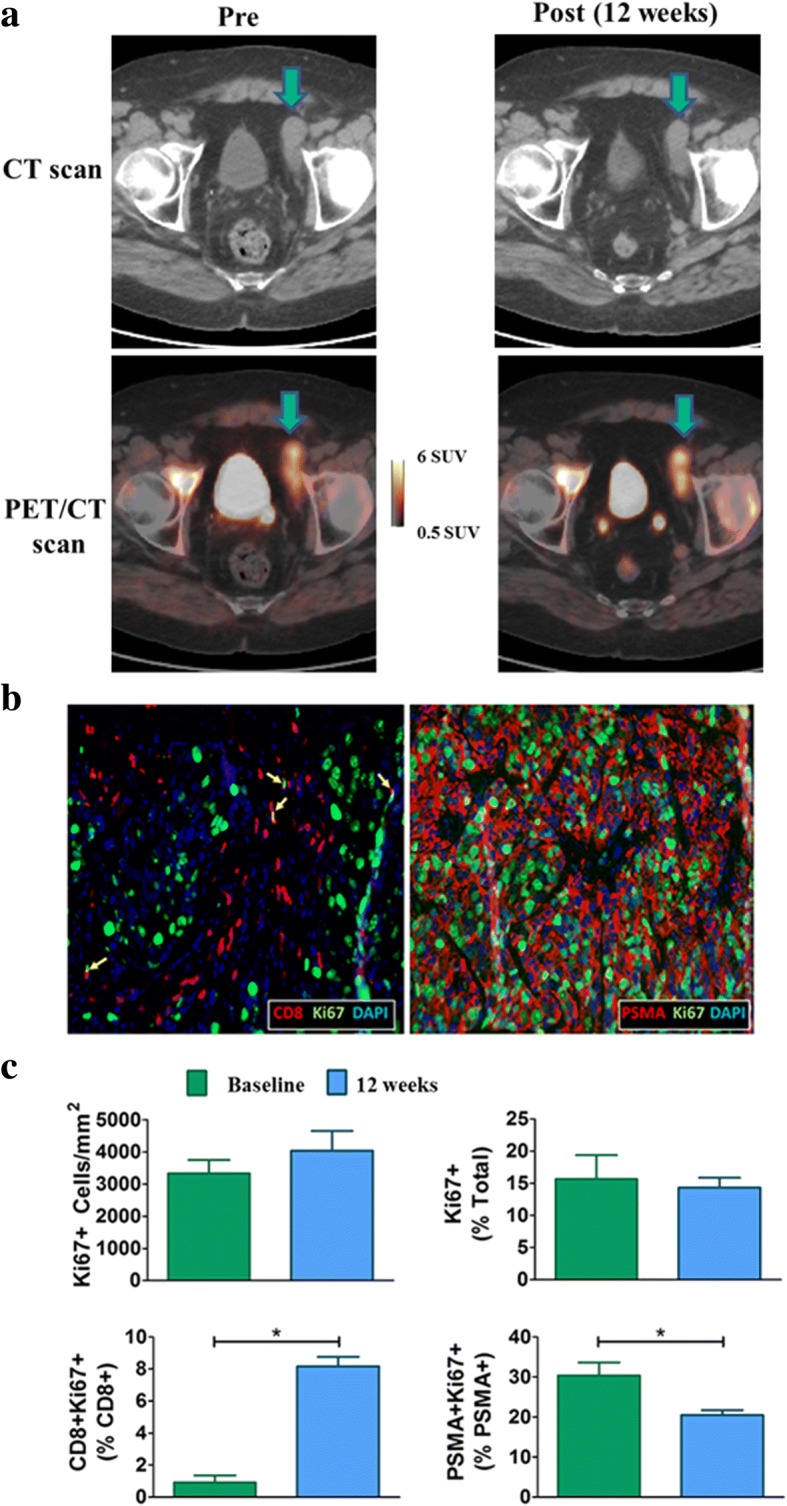
**a** Axial CT and PET/CT slices with a metastatic tumor indicated. At week 12 this patient had experienced diminished PSA and RECIST measurements but increased tumor FLT uptake. By week 16, this patient was found to have progressive disease with marked increases in tumor size and PSA. **b** Immunofluorescence images show representative FFPE sections taken from the week 12 biopsy of the tumor indicated in part (a). The left immunofluorescence image shows proliferating T cells (Ki67 + CD8+; yellow arrows) and the right image shows proliferating tumor cells (Ki67 + PSMA+). **c** Quantification of the immunofluorescence images from the tumor indicated in part (**a**). The top row shows changes in the number of proliferating cells per unit area (left) and changes in the percentage of proliferating cells (right). The bottom row shows percent changes in proliferating CD8+ T cells (left) and proliferating PSMA+ tumor cells (right). *P-value less than 0.05

Three patients had soft-tissue tumor biopsies. Of these 3 patients, only 1 had a successful baseline and 12-week biopsy (one patient had no tumor cells present in the follow-up biopsy, excluding it from the analysis; the other patient had a marked reduction in tumor size during treatment, making a biopsy unfeasible upon follow-up). The patient with successful baseline and 12-week tumor biopsy (patient #5) had the biopsies evaluated immunohistochemically for comparison with changes in other markers (Fig. 4). From baseline to 12 weeks, this patient’s PSA decreased 42%, sum of tumor diameters decreased 30% (RECIST measurement), and tumor FLT SUV_mean_ increased 10% (Fig. 4a). Immunofluorescence staining of this patient’s biopsy tissue revealed the majority of proliferating cells were prostate cancer cells at both baseline and follow-up (Fig. 4b). Quantification of the immunofluorescence images revealed a non-significant increase in the number of proliferating (Ki67+) cells per unit area from baseline to 12 weeks that is in agreement with the slight increase in FLT SUV_mean_ during this same time period (Fig. 4c). Notably, by week 16, this patient’s PSA had increased 26% and RECIST measurements had increased 31%, leading to classification of progressive disease.

### FLT PET and immune-related adverse events

**Fig. 5:**
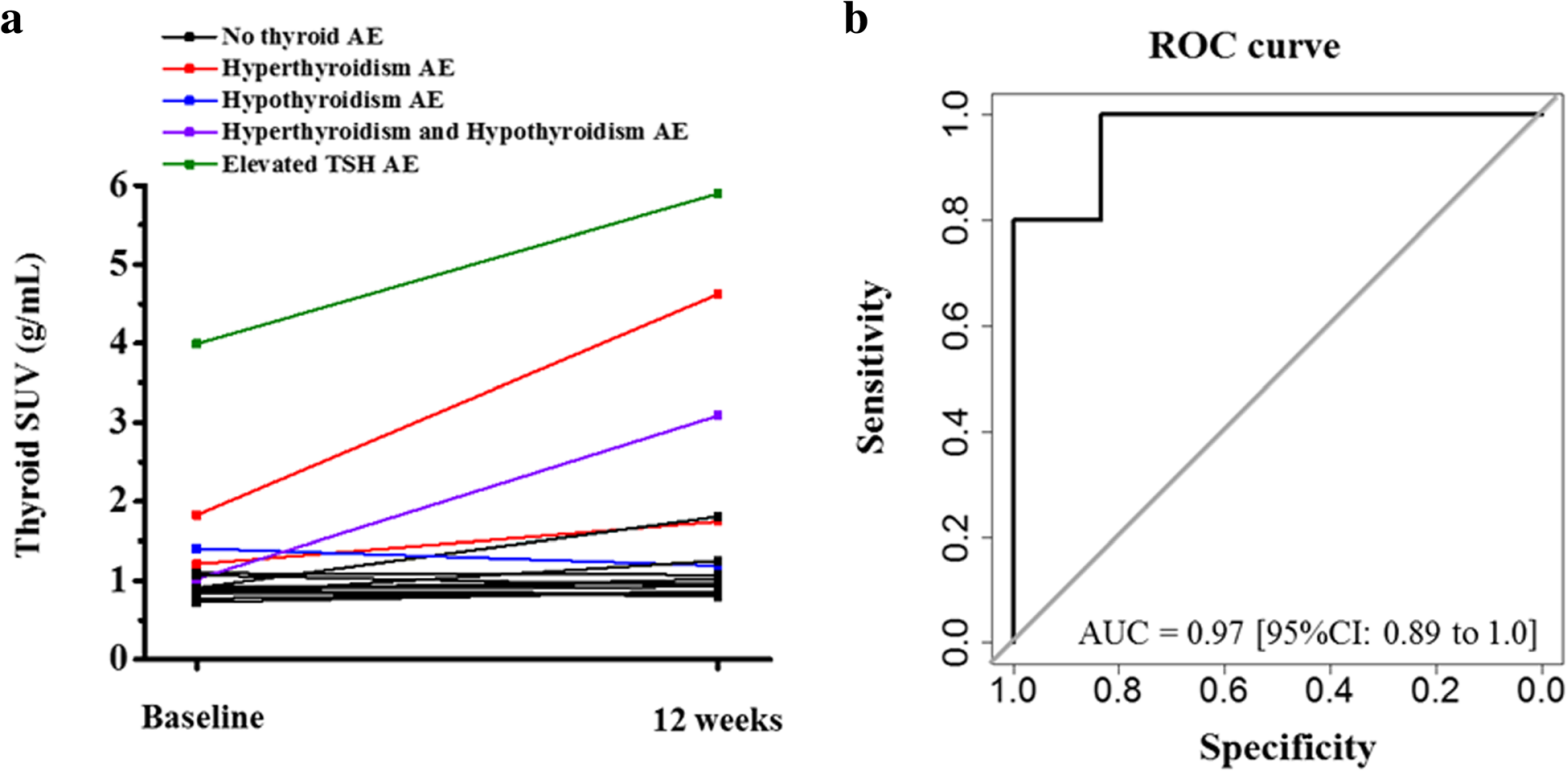
**a** Thyroid SUV_mean_ at baseline and after three months for all patients. Patients that experienced a thyroid related-adverse event of Gr2 or greater are shown in various colors to distinguish them from patients that did not experience a thyroid-related adverse event (black). **b** Receiver operating characteristic curve showing the value of thyroid SUV_mean_ at baseline for predicting which patients will go on to experience a thyroid-related adverse event

Five out of 17 patients (29%) experienced a grade 2 or greater adverse event relating to thyroid function (median time to adverse event was 9 weeks). Three out of the five patients that experienced a thyroid-related adverse event had the adverse event occur prior to the second FLT PET scan at 12 weeks, indicating the second PET scan may not be as useful for making predictions (Fig. 5a). However, baseline FLT uptake in the thyroid was significantly predictive of whether or not a patient would go on to experience a thyroid-related adverse event (AUC = 0.97; *P* < 0.01) (Fig. 5b).

One out of 17 patients (6%) had grade 2 or greater pancreatitis. This patient experienced a 30% increase in pancreas FLT SUV_mean_ from baseline to 12 weeks. However, other patients experienced even greater increases in pancreas FLT uptake and did not go on to experience any adverse events relating to the pancreas. In addition, baseline pancreatic FLT uptake was not significantly different in the patient that experienced pancreatitis than in the patients that did not experience pancreatitis.

One patient experienced grade 3 adrenal insufficiency 38 weeks after starting treatment; however, there was no evidence of abnormal FLT uptake on the baseline or 12-week FLT PET scans. No association was evident between immune-related adverse events and progression-free survival.

## Discussion

In this study we evaluated FLT uptake changes in various lymphoid organs including non-metastatic lymph nodes, spleen, and bone marrow. This rationale was based on the expectation that treatment with pTVG-HP DNA vaccine and pembrolizumab would elicit changes in immune cell proliferation. Specifically relating to lymph nodes, previous work by Aarntzen et al. showed increased FLT uptake in lymph nodes after a dendritic cell vaccine was injected intranodally in patients with melanoma [37]. Thus, in the current study, we hypothesized that similar increases in FLT uptake would be evident in vaccine-draining lymph nodes following intradermal injection of pTVG-HP vaccine. Significantly increased FLT uptake was found in left axillary lymph nodes (draining nodes) when compared with right axillary lymph nodes (non-draining nodes). This suggests that at least a subset of patients experience a regional immune response to pTVG-HP vaccine that is characterized by increased cell proliferation in vaccine draining lymph nodes after 12 weeks of therapy.

Previous work by Ribas et al. showed significantly increased splenic FLT uptake following treatment with tremelimumab in patients with metastatic melanoma [36]. The authors of that study hypothesized that releasing the CTLA-4 checkpoint on cell cycle in lymphocytes resulted in increased cell proliferation in the spleen. Similarly, in this study, significant increases in splenic FLT uptake were evident. These increases were greatest in arm 3, where patients received a combination of pTVG-HP vaccine and pembrolizumab every 3 weeks. Interestingly, these increases in splenic FLT uptake were inversely correlated with progression-free survival time i.e. patients with greater increases in splenic FLT uptake had shorter progression-free survival. Further investigation of this phenomenon may be critical to understanding why some patients do not respond well to therapy.

Measurements of tumor size derived from anatomic imaging modalities (e.g. computed tomography, magnetic resonance imaging, etc.) have proven essential in oncology, particularly for assessing tumor responses to cytotoxic therapy [47]. However, these modalities are less useful in the context of immunotherapy where changes in anatomic tumor size may not be indicative of response [1]. We hypothesized that changes in tumor cell proliferation as measured via FLT PET would be more strongly associated with progression-free survival than anatomic imaging modalities. This is supported by the results of this study, where changes in FLT PET after 12 weeks were more predictive of time to progression (based on concordance index values) than changes in tumor size measured via CT after 12 weeks or changes in PSA after 12 weeks. This suggests FLT PET may offer an earlier marker of response than prevailing methods of clinical assessment. Higher baseline SUV also correlated with shorter PFS (Additional file 2 Table S1). This likely reflects the prognostic value of FLT PET/CT in characterizing functional disease burden.

One of the three patients with soft tissue tumor biopsies had a successful baseline and follow-up biopsy that could be compared directly with FLT PET changes. Analysis of this patient’s biopsy samples found a non-significant increase in the number of Ki67+ cells per unit area that is in agreement with the 10% increase in this patient’s tumor SUV_mean_ from baseline to 12 weeks. The percentage of CD8+ T cells expressing Ki67 was found to increase after 12 weeks; however, the majority of proliferating cells stained positive for PSMA, suggesting that the bulk of the FLT uptake was due to proliferating prostate cancer cells as opposed to proliferating immune cells. This patient experienced disease progression at 16 weeks due to a 31% increase in RECIST measurement, suggesting the increased FLT uptake at week 12 may have been an early indication of disease progression.

In this study 5/17 (29%) patients experienced an adverse event grade 2 or greater related to thyroid function. One case led to discontinuation of treatment that was followed by subsequent disease progression. We discovered significantly higher FLT uptake at baseline in thyroids of patients that went on to experience a thyroid-related adverse event during treatment. These results indicate that prior to therapy, there is elevated cell proliferation in thyroids of patients who are likely to experience thyroid related adverse events. A previous study suggested PD-1 blockade may cause latent thyroid auto-immunity to become clinically detectable and lead to subsequent thyroid-related adverse events [48]. Further study might elucidate whether elevated FLT uptake in thyroid tissue at baseline is providing a measure of proliferating T cells that is linked with latent thyroid auto-immunity. In this study, monitoring of thyroid-related adverse events was done by measuring serum changes in thyroid function. The results of this work suggest FLT PET imaging might also have a role in monitoring/predicting adverse events related to thyroid function.

Thyroid-related adverse events have been documented during pembrolizumab treatment but not during pTVG-HP vaccinations, making it likely that the thyroid-related adverse events in this study were caused by the pembrolizumab [49, 50]. This is supported by the fact that no patients in this study experienced a thyroid adverse event before having a pembrolizumab injection. Interestingly, one patient had a noticeable increase in thyroid FLT uptake less than 24 h after receiving their first pembrolizumab injection (shown in Fig. 1h). This suggests that the auto-immune effects mediated by pembrolizumab may be detectable using FLT PET as early as 1 day following pembrolizumab injection.

In this study 1/17 (6%) patients experienced a grade 3 pancreatitis 30 weeks after starting treatment. This patient had increased pancreatic FLT uptake from baseline to 12 weeks; however, greater increases in pancreatic FLT uptake were evident in patients that did not go on to experience pancreatitis. This indicates FLT PET may have limited accuracy for predicting the occurrence of pancreatitis. Similarly, a grade 3 adrenal insufficiency was not evident on the baseline or f12-week FLT PET scans, suggesting limited accuracy for detecting such events.

It would be of great value for future studies to explore the optimal timing of follow-up FLT PET scans since this is currently an area of uncertainty. Likely the optimal timing of PET scanning will not only depend on the particular immunotherapy but also the specific process being measured. For example, in this study the rationale for choosing the 12-week follow-up PET was based on the expected time frame for immune activation to occur following pTVG-HP vaccination [8]. Nonetheless, some patients may have immune responses and meaningful changes in cell proliferation at earlier timepoints. As indicated, increases in thyroid FLT uptake were evident less than 1 day after injection of pembrolizumab. Perhaps a similar change in FLT uptake occurs in tumors with preexisting immune cell infiltrates that may offer an early marker of tumor response.

This study was limited in that bone marrow metastases were not evaluated using FLT PET/CT imaging. This is because of the high FLT uptake in non-diseased bone marrow that makes identification and segmentation of bone marrow metastases challenging. Interestingly, for patients with bone marrow metastases and soft-tissue metastases, changes in FLT PET uptake in soft-tissue metastases alone, were predictive of clinical outcome.

## Conclusions

In this exploratory study patients with metastatic prostate cancer were treated with pTVG-HP DNA vaccine and pembrolizumab. Increases in FLT uptake in the spleen and vaccine draining lymph nodes suggest increased immune cell proliferation in these tissues as a pharmacodynamic effect of treatment. Changes in FLT uptake in soft-tissue tumors were predictive of progression-free survival, indicating that changes in cell proliferation within tumors may offer an early measure of response. It was also found that baseline FLT uptake in the thyroid was predictive of the occurrence of thyroid-related adverse events. Together these results suggest FLT PET could be a potentially useful biomarker in immunotherapy, providing a relative early marker of tumor and immune responses.

## Additional file

Additional file 1 **Figure S1.** Study schedule. (DOCX 42 kb)

Additional file 2 **Table S1.** Baseline FLT SUVs, tumor size measurements, and PSA levels were included in Cox proportional hazards regression models to assess association with progression-free survival time. (DOCX 16 kb)

## Abbreviations

AUC: area under the (receiver operating characteristic) curve
CT: computed tomography
CTLA-4: cytotoxic T-lymphocyte associated protein 4
DNA: deoxyribonucleic acid
FFPE: formalin fixed paraffin embedded
FLT: 3′-Deoxy-3’-^18^F-fluorothymidine
PAP: prostatic acid phosphatase
PD-1: programmed death protein 1
PD-L1: programmed death-ligand 1
PET: positron emission tomography
PSA: prostate specific antigen
PSMA: prostate specific membrane antigen
RECIST: Response Evaluation Criteria in Solid Tumors
SUV: standardized uptake value
