# Supplementary figures and images for: FLT PET/CT imaging of metastatic prostate cancer patients treated with pTVG-HP DNA vaccine and pembrolizumab
Source: J Immunother Cancer. 2019 Jan 30;7:23. doi: 10.1186/s40425-019-0516-1 (PMC6354338; doi:10.1186/s40425-019-0516-1)

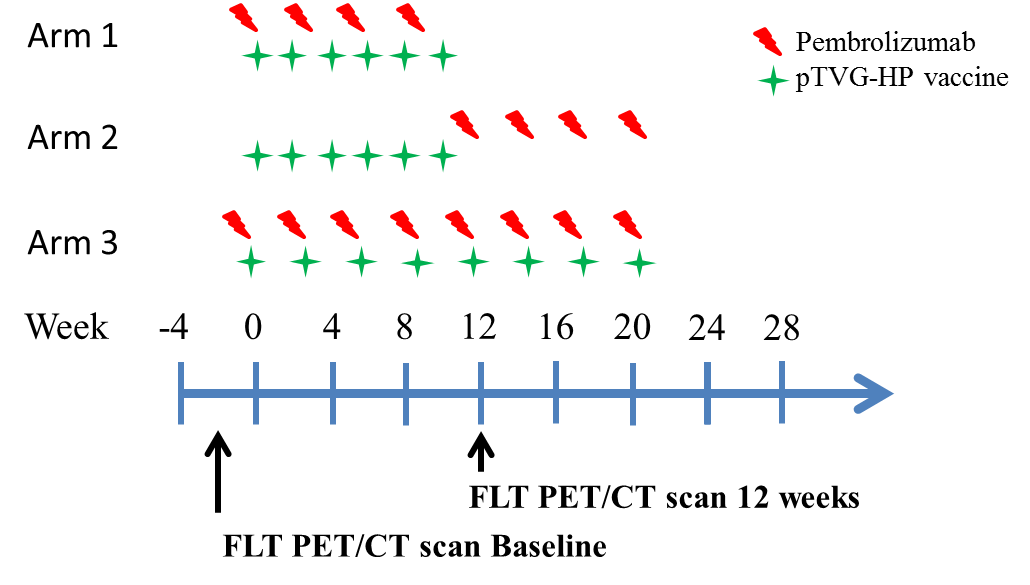

Supplement: Supplementary file 1 — Figure S1. Study schedule. (DOCX 42 kb) [file 40425_2019_516_MOESM1_ESM.docx]
