# Supplementary material for: FLT PET/CT imaging of metastatic prostate cancer patients treated with pTVG-HP DNA vaccine and pembrolizumab
Source: J Immunother Cancer. 2019 Jan 30;7:23. doi: 10.1186/s40425-019-0516-1 (PMC6354338; doi:10.1186/s40425-019-0516-1)
Supplement: Supplementary file 2 — Table S1. Baseline FLT SUVs, tumor size measurements, and PSA levels were included in Cox proportional hazards regression models to assess association with progression-free survival time. (DOCX 16 kb) [file 40425_2019_516_MOESM2_ESM.docx]

**Supplementary Table S1:** Baseline FLT SUVs, tumor size measurements, and PSA levels were included in Cox proportional hazards regression models to assess association with progression-free survival time.

|  | **Predictor** | **C-index^a^** | **C-index P value** | **Hazard Ratio** | **HR P value** | **N^b^** |
| --- | --- | --- | --- | --- | --- | --- |
| Traditional markers of response | PSA | 0.50  (0.29 to 0.71) | 1.0 | 0.74  (0.36 to 1.52) | 0.42 | 17  (12) |
|  | Soft tissue tumor size (RECIST) | 0.53  (0.30 to 0.76) | 0.78 | 0.96 (0.51 to 1.78) | 0.89 | 7  (6) |
| FLT PET uptake in lymphoid organs | Left axillary lymph node SUV_mean_ | 0.54  (0.43 to 0.76) | 0.69 | 0.71  (0.38 to 1.31) | 0.27 | 16  (11) |
|  | Spleen SUV_mean_ | 0.58  (0.39 to 0.77) | 0.40 | 0.83  (0.45 to 1.54) | 0.57 | 16  (11) |
|  | Bone marrow SUV_mean_ | 0.58  (0.45 to 0.72) | 0.24 | 1.07  (0.68 to 1.68) | 0.78 | 17  (12) |
| FLT PET uptake in tumors | Tumor SUV_mean_ | 0.76  (0.64 to 0.88) | <0.01 | 0.34  (0.11 to 1.10) | 0.07 | 10  (8) |
|  | Tumor SUV_total_ | 0.62  (0.50 to 0.74) | 0.04 | 1.61  (0.71 to 3.64) | 0.25 | 10  (8) |

^a^ C-index = concordance index (95% confidence interval shown in parenthesis)

^b^ N = number of patients included in calculation (value in parenthesis is number of patients that were not censored)
